# Degradation of progestagens by oxidation with potassium permanganate in wastewater effluents

**DOI:** 10.1186/1752-153X-7-84

**Published:** 2013-05-15

**Authors:** Paul B Fayad, Arash Zamyadi, Romain Broseus, Michèle Prévost, Sébastien Sauvé

**Affiliations:** 1 Department of Chemistry, Université de Montréal, Montreal, QC, Canada; 2 Department of Civil, Geological and Mining Engineering, École Polytechnique de Montréal, Montreal, QC, Canada

**Keywords:** Oxidation, Potassium permanganate, Steroid hormones, LDTD-APCI-MSMS, Endocrine disruptors, Kinetic rate constant

## Abstract

**Background:**

This study investigated the oxidation of selected progestagenic steroid hormones by potassium permanganate at pH 6.0 and 8.0 in ultrapure water and wastewater effluents, using bench-scale assays. Second order rate constants for the reaction of potassium permanganate with progestagens (levonorgestrel, medroxyprogesterone, norethindrone and progesterone) was determined as a function of pH, presence of natural organic matter and temperature. This work also illustrates the advantages of using a novel analytical method, the laser diode thermal desorption (LDTD-APCI) interface coupled to tandem mass spectrometry apparatus, allowing for the quick determination of oxidation rate constants and increasing sample throughput.

**Results:**

The second-order rate constants for progestagens with permanganate determined in bench-scale experiments ranged from 23 to 368 M^-1^ sec^-1^ in both wastewater and ultrapure waters with pH values of 6.0 and 8.0. Two pairs of progestagens exhibited similar reaction rate constants, i.e. progesterone and medroxyprogesterone (23 to 80 M^-1^ sec^-1^ in ultrapure water and 26 to 149 M^-1^ sec^-1^ in wastewaters, at pH 6.0 and 8.0) and levonorgestrel and norethindrone (179 to 224 M^-1^ sec^-1^ in ultrapure water and 180 to 368 M^-1^ sec^-1^ in wastewaters, at pH 6.0 and 8.0). The presence of dissolved natural organic matter and the pH conditions improved the oxidation rate constants for progestagens with potassium permanganate only at alkaline pH. Reaction rates measured in Milli-Q water could therefore be used to provide conservative estimates for the oxidation rates of the four selected progestagens in wastewaters when exposed to potassium permanganate. The progestagen removal efficiencies was lower for progesterone and medroxyprogesterone (48 to 87 %) than for levonorgestrel and norethindrone (78 to 97%) in Milli-Q and wastewaters at pH 6.0-8.2 using potassium permanganate dosages of 1 to 5 mg L^-1^ after contact times of 10 to 60 min.

**Conclusion:**

This work presents the first results on the permanganate-promoted oxidation of progestagens, as a function of pH, temperature as well as NOM. Progestagen concentrations used to determine rate constants were analyzed using an ultrafast laser diode thermal desorption interface coupled to tandem mass spectrometry for the analysis of water sample for progestagens.

## Background

Many endocrine-disrupting compounds (EDCs), such as steroid hormones (SH) have been detected in waste and surface water matrices [1-4]. They originate from naturally-occurring (e.g. normal urine excretion of estrogens from mammals) and synthetic (e.g. progestagens in oral contraceptives and hormone replacement therapy) sources. There has been growing concerns towards EDCs’ effects on the reproductive physiology of wildlife populations at very low concentrations, i.e. from 0.1 to 1.0 ng L^-1^[5-7]. Given their strong endocrine-disrupting potency and their occurrence, selected estrogens (estradiol, estrone and 17-α-ethinylestradiol) and progestagens (progesterone, levonorgestrel, medroxyprogesterone and norethindrone) have been targeted and detected in wastewater, surface water and drinking water [8-14].

Although the impact of natural and synthetic estrogens has been well documented, only a limited number of studies have been conducted on the ecotoxicological and environmental risk related to progestagens [15,16]. Progestagens are steroid hormones that produce effects similar to those of progesterone, a C-21 steroid hormone involved in the female menstrual cycle, pregnancy and the embryogenesis of humans and other species [17]. Several synthetic progestagens have been developed, given that natural progesterone is very rapidly inactivated in the human body. Synthetic progestagens can have various hormonal activities, such as estrogenic, anti-androgenic and androgenic [15]. Their presence in wastewater treatment plant (WWTP) effluents can pose a risk for the aquatic environment given their potential for impact on the reproductive success and the chemoreception of fish [18,19]. Synthetic progestagens were first developed and used in the 1960s as an effective method for contraception [20]. In Europe and America, the consumption rates of synthetic progestagens ranks as the first among all contraception methods, i.e. 33% in the United States and as high as 58% in some European countries [16]. In the United Kingdom, the estimated yearly usage of progestagens from oral contraception (~1723 kg/year) is higher than that of estrogens and androgens combined (~706 kg/year) [21]. In addition, the environmental amounts of progestagens and androgens excreted via urine is estimated to be 100 to 1000 times higher than that of estrogens in humans [22]. Therefore, progestagenic steroid hormones will be discharged into environmental waters from WWTP effluents and such releases should be better documented.

Several studies conducted in numerous countries [9,12,17], have shown that WWTP effluents and receiving water bodies contain sufficient amounts of progestagenic compounds to induce harmful effects on fish, with their concentrations varying from 0.2 to 205 ng L^-1^ for progestagens in wastewater samples [12,17,23]. With growing populations and increased discharge from WWTPs, the presence of progestagens in surface waters could be a cause for concern for drinking water treatment plants and therefore improved removal methods should be explored since conventional treatment methods have proven to be inadequate.

Permanganate [Mn(VII); KMnO_4_] is a relatively inexpensive and versatile oxidation agent with multiple applications in the degradation of multiples classes of contaminants, including phenolic and non-phenolic EDCs (dichlorvos, 4-t-butylphenol, estrone, triclosan and bisphenol-A) and various pharmaceuticals [24-28]. Permanganate may oxidize organic compounds through several reaction pathways, including electron exchange, hydrogen abstraction or direct donation of oxygen [29]. In acidic conditions, permanganate will decompose according to reaction (1), whereas in strong basic medium, reaction (2) will occur [30]:

(1) $${\mathrm{MnO}}_{4}^{-}+{4H}^{+}+{3e}^{-}\to {\mathrm{MnO}}_{2\left(s\right)}+2\;H_{2}O\;E^{0}=1.68V$$

(2) $${\mathrm{MnO}}_{4}^{-}+{2H}_{2}O+{3e}^{-}®{\mathrm{MnO}}_{2\left(s\right)}+{4\mathrm{OH}}^{-}\;E^{0}=0.60V$$

In contrast to other oxidants such as chlorine and ferrate (VI), permanganate is effective over a wide pH range and can control the formation of trihalomethanes and other disinfection by-products [30,31]. In addition, permanganate will generate an insoluble environmentally benign reduction product (MnO_2(s)_), which can enhance coagulation and simultaneously adsorb trace metals before their removal by sedimentation/filtration [27,32,33]. Previous work has established rate constants for selected estrogens (estradiol, estriol and estrone) using potassium permanganate, with values ranging from 16 to 38 300 M^-1^ s^-1^ at pH 5.0 to 12 [24,26]. Currently, no published information for the removal of progestagens by potassium permanganate is available. Progestagens should have different reactivity towards permanganate due to the absence of a phenolic moiety in their structures as compared to the estrogens.

Based on geology, climate, human influence and the surrounding watershed, different water sources will contain variable amounts of natural organic matter (NOM) with different characteristics, such as high and low molar mass organic material [34-36]. During the past 20 years, a significant increase in NOM concentrations has been observed in several surface water sources worldwide [37-40]. Permanganate has been shown to react with NOM compounds and should be considered when evaluating reaction rate constants [26,28]. The operation of water treatment facilities will therefore be influenced by NOM, since it can interfere with the oxidation removal of other contaminants by competitive consumption [41]. The impact of temperature (an especially relevant parameter for water utilities spawning their operation over four distinct seasons) and the dissolved fraction of NOM on kinetic rates have not yet been studied for the oxidation of progestagens with potassium permanganate.

To date, all analytical procedures used to determine oxidation rate constants for EDCs and steroid hormones have included the use of chromatography (liquid or gas) coupled to tandem mass spectrometry (MS/MS), ultraviolet or diode array detection [42]. Fayad et al. (2010) developed a sensitive method enabling high-throughput sample analysis of eight selected steroid hormones using a novel sample introduction method, LDTD-APCI coupled to MS/MS. The analysis time is achieved in seconds (<10 sec) compared to several minutes using the more traditional chromatography methods. As a result, the identification and quantification of the studied compounds will be much faster, the total analysis cost will be reduced and sample throughput will be significantly increased.

The objectives of this study were to: *i)* determine the rate constants for the reaction of potassium permanganate with progestagens (levonorgestrel, medroxyprogesterone, norethindrone and progesterone) in pure water and wastewater effluents using laboratory bench-scale assays, *ii)* evaluate the influence of operating conditions, i.e. pH, presence of NOM and temperature, on permanganate rate constants, *iii)* assess the validity of the determined rate constants when permanganate oxidation is applied to wastewaters. Furthermore, this work illustrates the advantages of using a novel analytical method, the LDTD-APCI-MS/MS apparatus, which is an alternative approach to chromatographic methods. This analytical approach facilitates the implementation of large experimental designs that require the evaluation of many different conditions and various time intervals. This method has allowed us to realize a quick determination of oxidation rate constants, while lowering analysis cost (no chromatography system is necessary) and increasing sample throughput. To the best of our knowledge, this paper presents the first results on the permanganate-promoted oxidation of steroid hormones without aromatic moieties, i.e. progestagens, as a function of pH, temperature as well as NOM levels.

## Experimental

### Chemicals

The selected steroid hormones (levonorgestrel (LEVO), medroxyprogesterone (MEDRO), norethindrone (NORE) and progesterone (PROG)) used for this study are listed in Additional file 1: Table S1 with their molecular structures presented in Additional file 1: Figure S1. All progestagen standards (purity ≥ 97%) were purchased form Sigma Aldrich (St. Louis, MO). Isotopically-labeled 17α-ethinylestradiol, [^13^C_2_]-EE2, was used as an internal standard (IS) and obtained from ACP Chemical Inc. (Montreal, QC, Canada). Other chemicals, including potassium permanganate (KMnO_4_), potassium phosphate (monobasic and dibasic) and ascorbic acid were of analytical grade and used without further purification. All solvents used were of HPLC grade purity from Fisher Scientific (Whitby, ON, Canada) and ultrapure water (18 MΩ cm) used was produced with a Milli-Q (Millipore, USA) apparatus.

Individual steroid hormone stock solutions were prepared in methanol (MeOH) at a concentration of 1000 mg L^-1^ and kept at −20°C in amber vials for a maximum of three months. A mixed steroid hormone working solution was prepared prior to the experiments at a concentration of 200 mg L^-1^ by dilution in MeOH of individual stock solutions aliquots for spiking solutions at the desired concentrations. Phosphate buffers of pH 6 and 8 (final concentrations: 50 mM) were prepared by dissolution of the commercial compounds in water. Potassium permanganate working solution (5.638 mmol L^-1^), ascorbic acid working solution (65.68 mmol L^-1^) and other reagents were also freshly prepared in Milli-Q water prior to the experiments and stored in amber bottles at 4°C.

### Water quality characterization

For the laboratory bench-scale experiments, wastewater effluents samples were taken from the water outlet of two municipal WWTPs in the province of Quebec, Canada. The first, WWTP A produces water with a DOC of 2.3 mg C L^-1^, an alkalinity of 80 mg CaCO_3_ L^-1^, UV absorbance (at 254 nm) of 0.027 cm^-1^, and a pH of 8.24. The second, WWTP B, produces water with a DOC of 7.7 mg C L^-1^, an alkalinity of 27 mg CaCO_3_ L^-1^, UV absorbance (at 254 nm) of 0.047 cm^-1^, and a pH of 6.30. Wastewater samples were not adjusted for pH and oxidation was carried out at ambient pH. The water samples were not analyzed for steroid hormones prior to oxidation experiments since their concentrations are orders of magnitude lower than the concentration spiked during the bench-scale experiments, as previously documented [43]. The water samples were collected in 10 L polypropylene carboys washed and rinsed successively with distilled and ultrapure water (Milli-Q). Wastewater effluent samples were filtered (0.45 μm polyethersulfone) and kept at 4°C prior to the oxidation experiments. Prior to DOC analyses, samples were passed through pre-rinsed (1 L ultrapure water) 0.45 μm cellulose nitrate Supor-450 membrane filters (PALL Life Sciences, USA). DOC measurements were made using a 5310C total organic carbon analyzer (Sievers Instruments Inc., USA).

### Analytical methods

The validation and optimization parameters of the LDTD-APCI-MS/MS method used for the detection and quantification of the selected steroids were previously described [44]. Briefly, water samples recuperated from the bench-scale oxidation experiments were first spotted (2 μL) into the LazWell 96-well polypropylene plate cavities and then left to dry (in a forced air oven at 30°C). Upon operation, a glass transfer tube is inserted into a well by an air-powered piston to avoid any sample loss. An infrared (IR) laser diode (980 nm, 20 W, continuous) is then focalized to impact the back of the metal inserts, thermally desorbing the dried sample which is vaporized into the gas phase. The uncharged analyte molecules travel along the transfer tube by a carrier gas to eventually reach the corona region for ionization by APCI and then be transferred to the MS inlet [44]. The MS/MS parameters for the selected steroid hormones are presented in Additional file 1: Table S2. Further details of the theory and principles behind the LDTD (Phytronix Technologies, Quebec, QC, Canada) are provided in the SI (Text S1).

In the kinetic experiments, permanganate was analyzed at 515 nm with a spectrophotometer (Varian-Cary 100, Victoria, Australia) in a 1-cm quartz cell, by the *N*, *N*-diethyl-*p*-phenylenediamine (DPD) colorimetric method [45].

### Laboratory bench-scale experiments

Oxidation experiments with potassium permanganate were conducted in 500 mL amber bottles (Fisher Scientific, Whitby, ON, Canda). All reactor assays were performed in duplicates. For ultrapure water (Milli-Q) samples, the pH was adjusted to 6.0 and 8.0 using the appropriate buffered phosphate solutions prior to the oxidation tests. It has been reported that phosphate buffers can enhance oxidation by permanganate of a certain number of phenolic compounds (i.e. triclosan, phenol, 2,4-dichlorophenol [46,47]). This is in contrast with several other studies performed on non-phenolic and phenolic compounds alike (e.g. chlorinated ethylenes, microcystins and carbamazepine) where phosphate buffer had no reported impact on oxidation mechanisms using permanganate [27,30,48]. There are no reported studies in the literature that would suggest that progestogenic-like steroid hormones (non-phenolic) would be influenced by the addition of phosphate buffer for permanganate oxidation. For WWTP A and WWTP B effluents samples, buffered solutions were not necessary since their natural pH values were 8.24 and 6.30, respectively. Phosphate buffers were only used for the assays using ultrapure water. The pH values in the reactors were constant throughout the oxidation tests with changes between the initial and final pH not exceeding 0.2.

In order to perform the oxidation experiments at different temperatures (from 5 ± 2°C to 30 ± 2°C), the reactors were placed in a temperature-controlled water bath. The initial concentrations of steroid hormones spiked in the reactors ranged from 0.883 to 9.76 μM, with 17 to 53 μM of permanganate added for the oxidation tests. Aliquots of 1.8 mL were collected at specific time intervals into a 2 mL amber vial containing a stoichiometric ratio of ascorbic acid to immediately quench the residual permanganate. It was previously established that ascorbic acid was suitable to quench oxidant residuals during sampling, while not affecting the stability of the studied ECDs [43]. Samples were then analyzed by LDTD-APCI-MS/MS to determine the residual concentration of steroid hormones.

The solvent used for the dissolution of the steroid hormones spiked in the reactors was MeOH. Initial tests were performed with acetonitrile and MeOH to assess their impact on permanganate decay rates in reactors with ultrapure water (without steroid hormones). Using MeOH showed that there was no decrease of initial permanganate concentration for up to 60 min contact times (Additional file 1: Figure S3). Reactor blank experiments (no steroid hormones added) for both wastewaters with the addition of permanganate were also conducted. All rate constants were determined under pseudo-first-order conditions, with permanganate in excess, in a batch reactor in ultrapure and wastewaters at pH 6.0-8.2.

### Determination of rate constants for the reaction potassium permanganate

The kinetics of the reactions of permanganate with organic and inorganic compounds is typically second order, i.e. first order with respect to the oxidizing agents (OAs) and the contaminant concentrations [49]. The degradation of steroid hormones by permanganate can be described with the following equation:

(3) $$-\frac{d\left[\mathrm{TC}\right]}{\mathrm{dt}}=k_{\mathrm{OA}}\;\left[\mathrm{OA}\right]\left[\mathrm{TC}\right]$$

Where TC = target compounds and *k*_OA_ = reaction rate constant for the applied oxidant. The rate constant is obtained from the integration of Eq. (3):

(4) $$\ln\;\left(\frac{{\left[\mathrm{TC}\right]}_{t}}{{\left[\mathrm{TC}\right]}_{o}}\right)=-k_{\mathrm{OA}}\int_{o}^{t}\left[\mathrm{OA}\right]\mathrm{dt}$$

where $\int_{o}^{t}\left[\mathrm{OA}\right]\mathrm{dt}$ is the time-integrated oxidant concentration. The value of the second-order rate constant can be found from the gradient of a plot of ln removal of the target compound against the time-integrated oxidant concentration. The latter defines an oxidant exposure (CT). In this study, CT values (mg.min L^-1^) were performed using the integrated CT concept [50], for which the effective CT at time t (min) is equal to the area under the decay curve at that time. CT values were calculated using the oxidant concentration profiles (Eq. (4)) and assuming a simple first-order decay:

(5) $${\mathrm{CT}}_{\mathrm{effective}}=\int C\left(t\right)\;\mathrm{dt}=\frac{C_{o}}{k'}\;\left[1-\exp\left(-k'\cdot t\right)\right]$$

where C = oxidant residual (mg L^-1^); C_o_ = initial oxidant residual (mg L^-1^) determined from the exponential fit of the relation between the oxidant residual and the time (min); k’ = oxidant first-order decay constant (min^-1^).

## Results and discussion

### Permanganate decay in ultrapure and wastewaters

Experimental decay data fit the first-order decay rates of permanganate (R^2^ ≥ 0.90) in ultrapure and effluent waters (Additional file 1: Figure S3). The values of apparent first-order rate constants for permanganate (k_OA_, sec^-1^), with and without the addition of steroid hormones (SH) were calculated by linear regression and resulting apparent rate constants are summarized in Table 1, in wastewaters at ambient pH 6.3 for WWTP B and pH of 8.2 for WWTP A. The rate constants increased slightly in the presence of SHs (1.2 and 1.13 × 10^-3^ sec^-1^) compared to wastewaters without the addition of SHs (0.57 and 0.44 × 10^-3^ sec^-1^), and were not influenced by pH (Table 1). The decay rates with KMnO_4_ were similar in ultrapure water and wastewaters in the presence of SHs at pH 6.3 and 8.2 (1.0-1.2 × 10^-3^ sec^-1^). This suggests that the influence of NOM did not further contribute to permanganate decay when compared to the presence of SHs.

**Table 1:** **First-order apparent rate constants (*k*, sec**^**-1**^**) for permanganate decay with and without progestagens in wastewaters at 22 ± 2°C for pH 6.3 (WWTP B, DOC 7.7 mg C L**^**-1**^**) and 8.2 (WWTP A, COD 2.3 mg C L**^**-1**^**)**^***a***^

	***k***_**KMO4**_**(×10**^**–3**^**sec**^**–1**^**)**	
	**Progestagens**	
*Wastewaters*	*pH 6.3*	*pH 8.2*
without SH	0.57 ± 0.02	0.44 ± 0.06
	(0.99)	(0.99)
with SH	1.2 ± 0.1	1.13 ± 0.07
	(0.94)	(0.98)
*Milli-Q water*	*pH 6.0*	*pH 8.0*
with SH	1.1 ± 0.2	1.0 ± 0.2
	(0.90)	(0.93)

^*a*^ Values in parentheses represent coefficients of determination (R^2^) and uncertainties on *k* values represent calculated standard deviation (SD) given from the linear regression equation.

### Oxidation of steroid hormones

**Figure 1:**
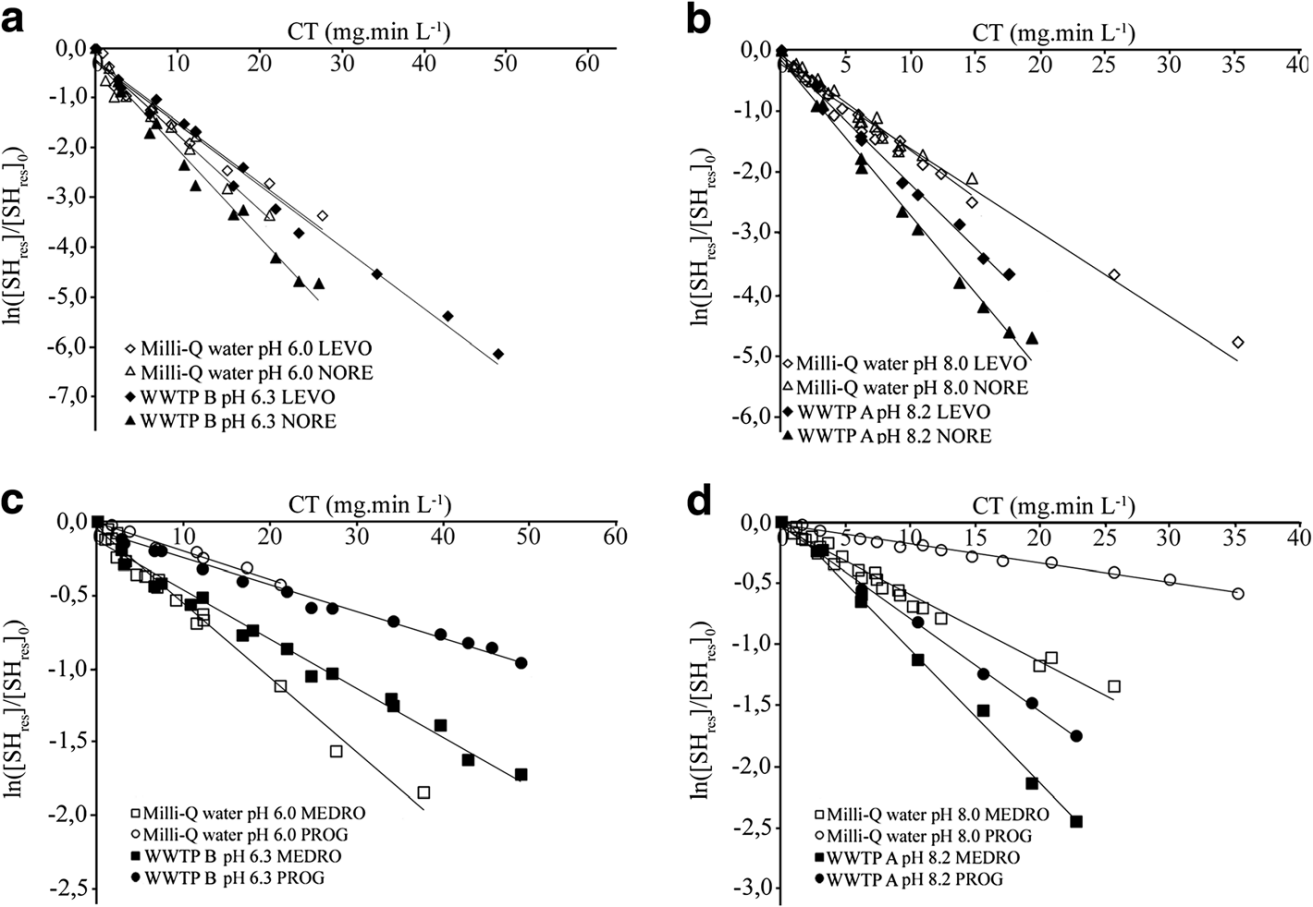
**Second-order rate kinetic plots for the oxidation of progestagenic steroid hormones (SH) by permanganate and the resulting effect of dissolved natural organic matter (NOM) from wastewaters (WWTP A and WWTP B) for progestagens at a) and c) pH 6.0, b) and d) pH 8.0 when compared to Milli-Q water according to progestagens having similar reaction rate constants.** Experimental conditions were, [KMnO_4_]_0_ from 17 to 39 μM and [progestagens]_0_ from 1.9 to 3.9 μM. Solid lines represent the linear regression of the measured data (symbols) with their related coefficients of determination (R^2^) ≥ 0.96.

Previous studies have investigated the reaction between oxidizing agents (permanganate and chlorine) with estrogenic steroid hormones [26,49]. They have shown that the reaction followed second-order kinetics overall, first-order with respect to estrogens and oxidation agents (OA). In determining the apparent first-order rate constants, the initial concentration of SH found in wastewaters was considered negligible (low ng L^-1^), about a thousand time lower than the spiked concentration used in batch reactors (~0.5 to 2 mg L^-1^). Additional file 1: Figure S3 shows that the decay of the four progestagens exposed to permanganate follows the second-order rate law expressed by Eq. (3). The tabulated first-order rate constants (Table 1) were derived from the dissolved permanganate residual decay curves in the presence of SH (Additional file 1: Figure S4) from which CT values were calculated using Eq. (5). The second-order rate constants (*k*_OA-SH_, M^-1^ sec^-1^) were then measured by linear regression of ln([SH_res_]/[SH_res_]_0_) as a function of CT (mg.min L^-1^), as shown in Figure 1. The oxidation experiments were conducted at 22 ± 2°C, and the calculated rate constants obtained from duplicate experiments are presented in Table 2. The standard deviations (SD) remained below 10% in all case with R^2^ ≥ 0.96 for all kinetic plots. The second order reaction rate constant for progestagens with permanganate varied from 23 (PROG) to 368 (NORE) M^-1^ sec^-1^ in Milli-Q and wastewaters at both pH values (6.0 and 8.0). Two pairs of progestagens exhibited similar reaction rate constants, i.e. PROG and MEDRO (23 to 80 M^-1^ sec^-1^ in Milli-Q water and 26 to 149 M^-1^ sec^-1^ in wastewaters, at pH 6.0 and 8.0) and LEVO and NORE (179 to 224 M^-1^ sec^-1^ in Milli-Q water and 180 to 368 M^-1^ sec^-1^ in wastewaters, at pH 6.0 and 8.0). The different ranges of kinetic decay rates could be related to the differences in their chemical structures and attack sites (Additional file 1: Figure S1). This similarity was also observed when ozone was applied to progestagens in Milli-Q water (pH 8.10) at 20°C, where PROG and MEDRO had rate constants of 601 M^-1^ sec^-1^ and 558 M^-1^ sec^-1^, respectively [43]. Several proposed mechanisms suggest that the potential sites of attack on the four progestagens by permanganate are the double bond between C4-C5, the hydroxyl group on C12 and the double-bonded oxygen on C3 (Additional file 1: Figure S1) [29,33]. The presence of the ethynyl groups on C12 for LEVO and NORE (Additional file 1: Figure S1) are reputed to react with permanganate [29] and could explain the higher rate constants we observed relative to PROG and MEDRO (Table 2). The very low rate constants measured for PROG could result from the absence of a hydroxyl function on the C12, lowering the potential attack sites for permanganate.

**Table 2:** **Steroid hormone (SH) oxidation rate constants (*k*, M**^**-1**^ **sec**^**-1**^**), removal efficiency (%) between 10 and 60 min and half-lives (*t***_***1/2***_**, min) derived from oxidation experiments using permanganate in Milli-Q and wastewaters (WWTP A, pH 8.2, DOC 2.3 mg C L**^**-1 **^**and WWTP B, pH 6.3, COD 7.7 mg C L**^**-1**^**) at 22 ± 2°C for pH 6.0 and 8.0**^***a***^

**Compounds**	**Milli-Q water**						**Wastewaters**					
	***k ***_**KMnO4-SH **_**(M**^**–1**^ **sec**^**–1**^**)**		**Removal (%)**		***t ***_**1/2 **_**(min)**		***k ***_**KMnO4-SH **_**(M**^**–1**^ **sec**^**–1**^**)**		**Removal (%)**		***t ***_**1/2 **_**(min)**	
	**pH 6.0**	**pH 8.0**	**pH 6.0**	**pH 8.0**	**pH 6.0**	**pH 8.0**	**pH 6.3**	**pH 8.2**	**pH 6.3**	**pH 8.2**	**pH 6.0**	**pH 8.0**
LEVO	179 ± 10	199 ± 6	89 ± 12	89 ± 12	5.6	5.0	180 ± 5	302 ± 9	97 ± 8	90 ± 1	5.6	3.3
	(0.96)	(0.98)					(0.96)	(0.98)				
MEDRO	73 ± 2	80 ± 2	57 ± 20	58 ± 12	14	12	49 ± 2	159 ± 4	71 ± 1	87 ± 4	2.1	6.3
	(0.98)	(0.98)					(0.98)	(0.99)				
NORE	219 ± 13	224 ± 8	90 ± 11	78 ± 3	4.6	4.5	257 ± 10	368 ± 11	96 ± 2	92 ± 1	3.9	2.7
	(0.96)	(0.97)					(0.98)	(0.98)				
PROG	28 ± 2	23 ± 1	48 ± 11	41 ± 5	36	44	26 ± 2	109 ± 4	58 ± 3	80 ± 20	38	9.2
	(0.97)	(0.98)					(0.98)	(0.98)				

^*a*^ Values in parentheses represent coefficients of determination (R^2^) and uncertainties on *k* values represent calculated standard deviations (SD) given from the linear regression equation. Uncertainties on removal values represent standard deviations (SD) between replicates (n = 2).

Table 2 also shows that pH did not have an impact on rate constants for progestagens either in ultrapure or wastewater effluents. This was expected since these compounds do not present any acid or basic character with predicted pK_a_ values between 17.0 and 19.3 for LEVO, NORE and MEDRO (Additional file 1: Table S1). The influence of dissolved organic matter was also investigated by using effluent wastewaters with different levels of NOM (WWTP A with 2.3 mg C L^-1^ and WWTP B with 7.7 mg C L^-1^) and conducting the oxidation experiments at ambient pH. With aromatic moiety being one of the main predicted sites of attack for permanganate, a competition effect is possible with background components in waters that contain electron rich moieties, such as humic acids [51]. Figure 1a and 1c as well as Table 2, show that at pH 6 the presence of NOM, regardless of the concentration present, did not have a significant effect on the rate constants with permanganate for LEVO, NORE and PROG (e.g. 179 to 180 M^-1^ sec^-1^ for LEVO, in Milli-Q (pH 6.0) and effluent waters (WWTP B, pH 6.3). On the other hand, oxidation of MEDRO was limited in effluent waters at pH 6.3. At higher pH, the presence of NOM systematically improved the efficiency of the oxidation of progestagens, as illustrated in Figure 1b and 1d as well as in Table 2.

Several ligands such as phosphate buffer, EDTA, and humic acid, have been shown to exert pH-dependent oxidation enhancement of phenolic compounds (Jiang et al. 2009) supporting the hypothesis of a catalytic role produced by the aqueous manganese intermediates (Mn(INT)_aq_) which are seemingly stabilized by metal-binding ligands [46]. This was illustrated with an enhancement of the oxidation of phenolic compounds that was also noted for the presence of NOM in river water and wastewaters and attributed to the formation of such unidentified Mn(INT)_aq_ species with a pH dependency of the r_ligand_ up to pH 9 [46].

The different trends observed in our data may reflect the fact that the role of ligands in Mn-promoted oxidation is closely related to the structure of the target organic molecules, as proposed by Jiang et al. [46]. A significant improvement of oxidation of phenolic compounds using humic acids of various origins has been reported [46,47,52]. One could hypothesize that the presence of NOM in this case also contributes to enhance the oxidation of the progestagens. Hu et al. [27] have attributed the degradation efficiency of the oxidation of carbamazepine in the presence of permanganate to an electrophilic attack, the stabilization of Mn(INT)_aq_ could also help improve this electrophilic attack onto the molecules of progestagens. In the case of our target compounds, NOM did not enhance oxidation at a pH 6.3 while a significant and consistent enhancement was noted at pH 8. Hu et al. did not observe any enhancement of carbamazepine oxidation at pH 5, 6 and 7 in the presence of 10 and 20 mg L^-1^ of humic acids [27]. Within our dataset, it is difficult to fully dissociate what proportion of the difference in efficiency between pH values of 6 and 8 is attributable to an effect due an unknown difference in the chemistry of both effluent wastewaters tested. With the presumption that the effect is solely attributable to the pH difference, the nature of the NOM as a function of pH could also partly explain the observed results.

Dissolved humic substances have been known to influence removal of organic compounds from municipal and industrial wastewaters [53,54] as well as being responsible for capturing and transporting both nonpolar (such as p,p’-DDT, 2,4,5,2’,5’-PCB and 2,4,4’-PCB) and polar (pesticides) contaminants [55,56]. This could be a result of the humic macromolecules that have been shown to be densely coiled at lower pH values, such as that of the WWTP B (pH = 6.3) effluent (Figure 1a and 1c) for progestagens, as depicted by the random coil model [57]. Therefore, *i)* the affinity of NOM to complex and stabilize Mn(INT)_aq_ is lowered and the resulting catalytic activity is no longer observed and *ii)* progestagens (log K_ow_ ≥ 2.97, Additional file 1: Table S1) could be complexed by the humic macromolecules in their hydrophobic structural voids cavities [58] making them less available for oxidation by permanganate. Inversely, at more alkaline pH, such as that of the WWTP A (pH = 8.2) effluent (Figure 1b and 1d) for progestagens, there is formation of negatively charged carboxyl groups found in the humic macromolecules. This induces mutual repulsion and expansion of the formerly coiled macromolecules [59], making them more flexible and slightly more polar in nature, thus promoting its reactivity allowing for the formation of more stable Mn(INT)_aq_ compounds while reducing the progestagens affinity with the NOM. The impact of the structure of the target compound, the nature of the ligand and the pH dependency of NOM on the kinetics of permanganate oxidation needs to be further investigated.

As the presence of NOM generally had little impact (at pH values around 6) or enhanced oxidation of the four progestagenic compounds tested (at pH values around 8), reaction rates measured in Milli-Q water could therefore be used by operators as a conservative estimate to predict the oxidation of the four selected progestagens in natural source waters when exposed to permanganate. It could be presumed that under favorable conditions (alkaline pH and organic ligands) the oxidation can be enhanced relative to than predicted in pure water.

### Effect of temperature on kinetic rate constants

**Figure 2:**
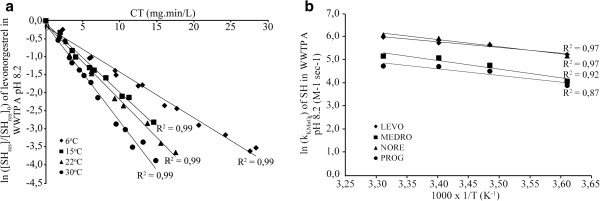
**Effect of temperature on the second-order decay of progestagens: a) levonorgestrel and b) its impact on measured rate constants for oxidation of progestagenic steroid hormones (SH) by permanganate.** The experimental conditions were, [KMnO_4_]_0_ from 38 to 53 μM, [progestagens]_0_ from 2.1 to 4.1 μM in wastewater (WWTP A) at pH 8.2 and temperature ranging from 6 to 30°C. Solid lines represent the linear regression of the measured data (symbols) with their related coefficients of determination (R^2^) also given.

Temperature dependence of the reaction between permanganate and progestagens was determined by measuring the rate constants at four different temperatures (6, 15, 22 and 30°C), at pH 8.0 (WWTP A) in wastewater. The effect of temperature on second-order rate constant plots is illustrated for LEVO in Figure 2, with the reaction rates increasing as a function of temperature. The corresponding second-order rate constants (Table 3) were used to calculate the activation energy by means of the linearized Arrhenius equation (Eq. (6)).

(6) $$\ln\;\left(K_{\mathrm{KMnO}4-\mathrm{SH}}\right)=\ln\;A-\frac{E_{a}}{\mathrm{RT}}$$

with k the rate constant, *E*_a_ the experimental energy of activation, R the gas constant, T the absolute temperature (K) and A the frequency factor. After linear regression analysis (R^2^ ≥ 0.87), the apparent activation energies (*E*_a_), ranging from 20 to 31 KJ mol^-1^, were calculated (Table 3). To the best of our knowledge, there are no published values for temperature dependent rate constants for progestagens. These *E*_a_ are lower than reported earlier for the oxidation of E1 by permanganate (43.07 KJ mol^-1^) [24], while comparable to other organic compounds, such as cyanotoxins (28.8 and 20 KJ mol^-1^) [30,60] and chloroethylene (24.4 and 39.1 KJ mol^-1^) [61,62]. According to the activation energy values measured, a temperature increase of 10°C will raise the oxidation rate by a factor between 1.4-1.6. As a result, the impact of temperature could be a relevant parameter for water utilities where oxidation removal potential could be optimized according to weather conditions.

**Table 3:** **Temperature effect on first-order apparent rate constants (*k*, sec**^**-1**^**), oxidation rate constants (*k*, M**^**-1**^ **sec**^**-1**^**) and activation energy (E**_**a**_**, KJ mol**^**-1**^**) for permanganate decay with and without progestagens (SH) in wastewater at pH 8.2 (WWTP A)**^***a***^

**Compounds**	**Natural waters WWTP A (pH 8.2)**								
	***k ***_**KMnO4 **_**(×10**^**–4**^ **sec**^**–1**^**) without SH**				***k ***_**KMnO4-SH **_**(M**^**–1**^ **sec**^**–1**^**)**				**E**_**a **_**(KJ mol**^**-1**^**)**
	**5°C**	**15°C**	**22°C**	**30°C**	**5°C**	**15°C**	**22°C**	**30°C**	
LEVO					184 ± 5	278 ± 9	302 ± 9	389 ± 13	20 ± 2
					(0.99)	(0.98)	(0.99)	(0.98)	
MEDRO					58 ± 1	115 ± 3	159 ± 4	171 ± 5	31 ± 2
	1.9 ± 0.2	1.8 ± 0.8	4.4 ± 0.6	5.4 ± 0.2	(0.99)	(0.99)	(0.99)	(0.98)	
	(0.98)	(0.82)	(0.99)	(0.94)	172 ± 8	288 ± 12	368 ± 11	429 ± 13	26 ± 2
					(0.97)	(0.98)	(0.99)	(0.98)	
PROG					48 ± 1	89 ± 2	109 ± 3	112 ± 3	23 ± 7
					(0.99)	(0.99)	(0.93)	(0.99)	

^*a*^ Values in parentheses represent coefficients of determination (R^2^) and uncertainties on k values represent calculated standard deviation (SD) given from the linear regression equation.

### Removal efficiencies and half-life

The elimination of SH by permanganate in Milli-Q and in wastewaters for pH 6.0 and pH 8.0, was investigated and values of removal percentage and half-life are summarized in Table 2.

**Figure 3:**
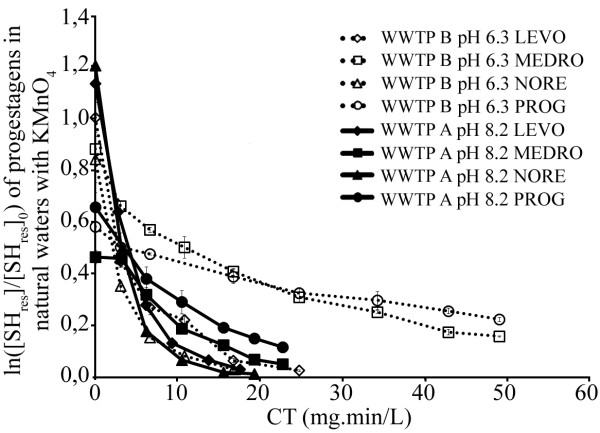
**Steroid hormone (SH) decay curves following oxidation with permanganate in wastewaters (WWTP A, pH 8.2 and WWTP B, pH 6.3) for progestagens.** [KMnO_4_]_0_ ranged from 17 to 39 μM. Solid (WWTP A) and doted (WWTP B) lines represent the trend of the measured data (symbols).

Steroid hormone decay curves following oxidation with permanganate in wastewaters (WWTP A, pH 8.2 and WWTP B, pH 6.3) for progestagens are presented in Figure 3. Removal efficiencies with permanganate were not affected by pH or dissolved NOM, with values from Milli-Q and wastewater effluents being similar (considering standard deviations). The removal of progestagens was lower for both pairs (similar chemical structures) PROG and MEDRO than LEVO and NORE, i.e. 48 to 87% with half-lives between 2.1 and 44 min compared to 78 to 97% with half-lives between 2.7 and 5.6 min, in Milli-Q and wastewaters at pH 6.0-8.2, respectively. This could be attributed to the presence of the ethynyl groups on C12 for LEVO and NORE (Additional file 1: Figure S1). In the case of PROG and MEDRO, a positive impact of dissolved NOM is observed with lower half-lives and higher degradation rates in wastewaters than in Milli-Q water, especially at pH 8.0. This background matrix of wastewaters has been shown to accelerate the oxidation of estrogens by permanganate [26]. These values were measured under typically-applied permanganate dosages (1 to 5 mg L^-1^) with a minimum contact time of 10 min and maximum of 60 min. A CT value of 25 mg.min L^-1^ is needed to reduce concentration by 94 and 99% for LEVO and NORE, between 67 and 94% for MEDRO, whereas for PROG the removal ranged from 59 to 87%, at both pH 6.0 and 8.0 in Milli-Q and natural waters. The results for progestagens show that LEVO, NORE and MEDRO are most susceptible to permanganate attack with even better results in wastewater effluents, especially for MEDRO at pH 8.0. PROG is less affected by permanganate and would require very long contact times for sufficient removal to occur which would not be representative of realistic operating conditions for typical water treatment plants.

## Conclusion

Oxidation kinetics of four selected progestagens (progesterone, medroxyprogesterone, norethindrone and levonorgestrel) with permanganate were investigated as a function of pH, NOM and temperature. The second-order rate constants ranged for 23 to 224 M^-1^ sec^-1^ in Milli-Q water and 26 to 368 M^-1^ sec^-1^ in wastewater, at pH 6.0 and 8.0, respectively. It was found that a variation in pH did not significantly influence the rate constant. The impact of NOM at typical ranges found in wastewater effluents was also minimal with a slight enhancement of oxidation at pH values around 8. The removal of progestagens was lower for both progesterone and medroxyprogesterone than for levonorgestrel and norethindrone, i.e. 48 to 87% with half-lives between 2.1 and 44 min compared to 78 to 97% with half-lives between 2.7 and 5.6 min, in Milli-Q and wastewaters at pH 6.0-8.2, respectively.

This work demonstrates the potential of permanganate to oxidize progestagens in drinking water within the typical ranges of operational values of pH and NOM. Proper attention should be given to provide adequate CT values in order to ensure the efficient removal of progestagenic steroid hormones and recalcitrant analogues. Further kinetic studies targeting the identification and environmental impact of possible by-products related to progestagens reaction with permanganate appear warranted.

## Competing interests

The authors declare that they have no competing interests.

## Authors’ contributions

PBF performed the main part of the experiments and drafted the manuscript. AZ and RB helped in the analysis of the data obtained, the drafting of the manuscript from oxidation experiments and assisted in performing the oxidation treatment bench-scale assays, respectively. MP and SS helped design the experiment, interpret and evaluate the results as well as revise the manuscript’s writing. All the authors read and approved the final manuscript.

## Supplementary Material

Additional file 1: Text S1 LDTD-APCI Source Principles. **Figure S1.** Molecular structures of selected steroid hormones and their acronyms, with atom numbering for the base structure of steroid hormones. **Figure S2.** Impact of solvents (methanol, MeOH and acetonitrile, ACN) on permanganate decay in batch reactor conditions without the addition of steroid hormones. Experimental conditions were, [KMnO_4_]_0_ = 10 mg/L at pH 8.0 in Milli-Q water with a 0.2% v/v addition of solvent into 500 mL batch reactor. Error bars represent the standard deviation of replicate measurements/batch (n = 3). **Figure S3.** Pseudo-first-order kinetic plots corresponding to decay curves of permanganate with progestagens (LEVO, MEDRO, NORE and PROG) in ultrapure and wastewater effluents according to pH values (WWTP A, pH 8.2 and WWTP B, pH 6.3). Experimental conditions were, [KMnO4]0 from 17 to 39 μM and [progestagens]0 from 1.9 to 3.9 μM. Solid lines represent the linear regression of the measured data (symbols) with their related coefficients of determination (R2) also given. **Figure S4.** Permanganate decay curves of a) permanganate with estrogens and b) permanganate with progestagens in Milli-Q and natural (WWTP A, pH 8.2 and WWTP B, pH 6.3) waters according to pH values. Experimental conditions were, [KMnO_4_]_0_ from 17 to 39 μM and [progestagens]_0_ from 1.9 to 3.9 μM. Solid and doted lines represent the trend for the measured data (symbols). **Table S1.** Physicochemical Properties of Selected Steroid Hormones. **Table S2.** MS/MS Parameters for the Analysis of Selected Steroid Hormones Analytes in Both Negative (NI) and Positive (PI) Ionization Mode by LDTD-APCI-MSMS. Click here for file
