# Supplementary material for: Degradation of progestagens by oxidation with potassium permanganate in wastewater effluents
Source: Chem Cent J. 2013 May 15;7:84. doi: 10.1186/1752-153X-7-84 (PMC3671215; doi:10.1186/1752-153X-7-84)
Supplement: Additional file 1: Text S1 — LDTD-APCI Source Principles. Figure S1. Molecular structures of selected steroid hormones and their acronyms, with atom numbering for the base structure of steroid hormones. Figure S2. Impact of solvents (methanol, MeOH and acetonitrile, ACN) on permanganate decay in batch reactor conditions without the addition of steroid hormones. Experimental conditions were, [KMnO4]0 = 10 mg/L at pH 8.0 in Milli-Q water with a 0.2% v/v addition of solvent into 500 mL batch reactor. Error bars represent the standard deviation of replicate measurements/batch (n = 3). Figure S3. Pseudo-first-order kinetic plots corresponding to decay curves of permanganate with progestagens (LEVO, MEDRO, NORE and PROG) in ultrapure and wastewater effluents according to pH values (WWTP A, pH 8.2 and WWTP B, pH 6.3). Experimental conditions were, [KMnO4]0 from 17 to 39 μM and [progestagens]0 from 1.9 to 3.9 μM. Solid lines represent the linear regression of the measured data (symbols) with their related coefficients of determination (R2) also given. Figure S4. Permanganate decay curves of a) permanganate with estrogens and b) permanganate with progestagens in Milli-Q and natural (WWTP A, pH 8.2 and WWTP B, pH 6.3) waters according to pH values. Experimental conditions were, [KMnO4]0 from 17 to 39 μM and [progestagens]0 from 1.9 to 3.9 μM. Solid and doted lines represent the trend for the measured data (symbols). Table S1. Physicochemical Properties of Selected Steroid Hormones. Table S2. MS/MS Parameters for the Analysis of Selected Steroid Hormones Analytes in Both Negative (NI) and Positive (PI) Ionization Mode by LDTD-APCI-MSMS. [file 1752-153X-7-84-S1.docx]

**Degradation of Progestagens by Oxidation with Potassium Permanganate in Waste Water Effluents**

**Supplementary Information**

Paul B. Fayad^1^, Arash Zamyadi^2^, Romain Broseus^2^, Michèle Prévost^2^ and Sébastien Sauvé^1*^

^1^Department of Chemistry, Université de Montréal, Montreal, QC, Canada

^2^Department of Civil, Geological and Mining Engineering, École Polytechnique de

Montréal, Montreal, QC, Canada

* Corresponding author.

Phone: 514-343-6749. Fax: 514-343-7586. E-mail: sebastien.sauve@umontreal.ca.

This file is 12 pages and contains 4 figures and 2 tables.

**Table of content:**

***Text S1-****Page S-3 to S-4.* LDTD-APCI Source Principles.

***Figure S1*-** *Page S-5.* Molecular structures of selected steroid hormones and their acronyms, with atom numbering for the base structure of steroid hormones.

***Figure S2*-***Page S-6.* Impact of solvents (methanol, MeOH and acetonitrile, ACN) on permanganate decay in batch reactor conditions without the addition of steroid hormones. Experimental conditions were, [KMnO_4_]_0_ = 10 mg/L at pH 8.0 in Milli-Q water with a 0.2% v/v addition of solvent into 500 mL batch reactor. Error bars represent the standard deviation of replicate measurements/batch (n=3).

***Figure S3*-*Page S-7.*** Pseudo-first-order kinetic plots corresponding to decay curves of permanganate with progestagens (LEVO, MEDRO, NORE and PROG) in ultrapure and wastewater effluents according to pH values (WWTP A, pH 8.2 and WWTP B, pH 6.3). Experimental conditions were, [KMnO4]0 from 17 to 39 µM and [progestagens]0 from 1.9 to 3.9 µM. Solid lines represent the linear regression of the measured data (symbols) with their related coefficients of determination (R2) also given.

***Figure S4-****Page S-8.* Permanganate decay curves of a) permanganate with estrogens and b) permanganate with progestagens in Milli-Q and natural (WWTP A, pH 8.2 and WWTP B, pH 6.3) waters according to pH values. Experimental conditions were, [KMnO_4_]_0_ from 17 to 39 µM and [progestagens]_0_ from 1.9 to 3.9 µM. Solid and doted lines represent the trend for the measured data (symbols).

***Table S1-*** *Page S-9.* Physicochemical Properties of Selected Steroid Hormones.

***Table S2-****Page S-10.* MS/MS Parameters for the Analysis of Selected Steroid Hormones Analytes in Both Negative (NI) and Positive (PI) Ionization Mode by LDTD-APCI-MSMS.

**Text S1.** **LDTD-APCI Source Principles.** The Arrhenius plots comparing the logarithm of the kinetic rates of the temperature dependencies of vaporization (dissociation of intermolecular bonds) and molecular decomposition (dissociation of intramolecular bonds) against the inverse temperature (1/T) would identify an intersection point at some value of 1/T, above which the vaporization of the uncharged molecular species would be favored and below which decomposition would be observed [1,3]. The heating rate of the LDTD laser is 3000°C/sec which allows the samples to be quickly heated at high temperatures, minimizing the time spent in the decomposition region and favoring vaporization which generates a greater amount of the uncharged molecular species.

The characterization of the thermal desorption processes associated with rapid sample heating and thin film deposition for the LDTD-APCI source have been shown on prednisone, a corticosteroid steroid hormone, that decomposed at a temperature of 234°C but was fully analyzed by LDTD-APCI-MS/MS at a desorption temperature of 170°C. Also sulfadiazine, a sulfonamide antibiotic, shows a desorption temperature observed between 95°C and 140°C though its bulk melting point is 252-256°C [10]. Unlike traditional LC-APCI, the LDTD-APCI ionization is performed in the absence of solvent reacting molecules as no liquid phase is introduced into the corona discharge region. Similarly to classical chemical ionization, the species present in the APCI region of the LDTD originate from gas-phase reactions involving proton transfer, governed by proton affinity in positive mode (PI) and gas-phase acidity in negative mode (NI), as well as charge exchange [6]. The solvent used for analyte deposition in LDTD-APCI will not induce signal suppression due to competition for protonation as can be the case in classical LC-APCI mobile-phase components [2,5,8]. In the absence of any mobile phase, it is the water traces in the carrier gas that will generate ionization in LDTD-APCI, since proton transfer in the PI mode mostly occurs between water cluster, H_3_O^+^(H_2_O)_n_ where n = 0-4 [13], and more basic analytes (higher proton affinity). Higher efficiency protonation in LDTD-APCI is accomplished in the presence of the smallest hydronium species because of their low proton affinity, i.e. H_3_O^+^ and (H_2_O)H_3_O^+^. Larger water clusters will form when the humidity (water saturation) in the ionization region is increased and negatively impact the analyte ionization process [11]. The larger hydronium cluster, with higher proton affinity, will affect sensitivity towards low proton affinity molecules and decrease their fragmentation because of an excess of energy in protonation reactions [12]. The main advantage of using APCI with the LDTD is that it is less susceptible to matrix effects and ionization suppression than with electrospray ionization [9].

O

O

H

H

H

P

r

o

g

e

s

t

e

r

o

n

e

(

P

R

O

G

)

O

O

H

H

H

M

e

d

r

o

x

y

p

r

o

g

e

s

t

e

r

o

n

e

(

M

E

D

R

O

)

O

H

C

H

3

O

H

H

H

L

e

v

o

n

o

r

g

e

s

t

r

e

l

(

L

E

V

O

)

O

H

O

H

H

H

N

o

r

e

t

h

i

n

d

r

o

n

e

(

N

O

R

E

)

O

H

**Figure S1** Molecular structures of selected steroid hormones and their acronyms, with atom numbering for the base structure of steroid hormones.

 **Figure S2** Impact of solvents (methanol, MeOH and acetonitrile, ACN) on permanganate decay in batch reactor conditions without the addition of steroid hormones. Experimental conditions were, [KMnO_4_]_0_ = 10 mg/L at pH 8.0 in Milli-Q water with a 0.2% v/v addition of solvent into 500 mL batch reactor. Error bars represent the standard deviation of replicate measurements/batch (n=3).

**

**

**Figure S3** Pseudo**-**first-order kinetic plots corresponding to decay curves of permanganate with progestagens (LEVO, MEDRO, NORE and PROG) in ultrapure and wastewater effluents according to pH values (WWTP A, pH 8.2 and WWTP B, pH 6.3). Experimental conditions were, [KMnO_4_]_0_ from 17 to 39 µM and [progestagens]_0_ from 1.9 to 3.9 µM. Solid lines represent the linear regression of the measured data (symbols) with their related coefficients of determination (R^2^) also given.

**

**

**Figure S4** Permanganate decay curves of permanganate with progestagenic steroid hormone (SH) in ultrapure and wastewater effluents (WWTP A, pH 8.2 and WWTP B, pH 6.3) waters according to pH values. Experimental conditions were, [KMnO_4_]_0_ from 17 to 39 µM and [progestagens]_0_ from 1.9 to 3.9 µM. Solid and doted lines represent the trend for the measured data (symbols).

**Table S1 Physicochemical Properties of Selected Steroid Hormones**

| **Steroid**  **Hormones** | **MW^a^**  **(g mol^-1^)** | **Water solubility^a^**  **(mg L^-1^ at 20^o^C)** | **Vapor pressure^a^**  **(mm Hg)** | **Log K_ow_^a^** | **pK_a_^b^** |
| --- | --- | --- | --- | --- | --- |
| PROG | 314.5 | 8.8 | 1.3 × 10^-6^ | 3.87 | ---- |
| LEVO | 312.5 | 2.1 | 3.9 × 10^-10^ | 3.48 | 19.3 |
| NORE | 298.4 | 7.0 | 7.3 × 10^-9^ | 2.97 | 19.3 |
| MEDRO | 344.5 | 2.9 | 1.9 × 10^-10^ | 3.50 | 17.6 |

^a^ The data was collected from the SRC PhysProp database. <http://www.syrres.com> [7]

^b^ The pK_a_ values for LEVO, NORE and MEDRO are predicted values listed according to the drug bank database. <http://www.drugbank.ca> [4]

**Table S2 MS/MS Parameters for the Analysis of Selected Steroid Hormones Analytes and Standards in Both Negative (NI) and Positive (PI) Ionization Mode by LDTD-APCI-MSMS**

**Compound**

**Ionisation**

**Precursor ion**

**Product ion**

**Relative intensity ratio**

**a**

**TL**

**CE**

**mode**

**(m/z)**

**(m/z)**

**(%)**

**(V)**

**(eV)**

EE2-

13

C

2

PI

281 [M-H

2

O+H]

+

133

100

48

23

159

22 ± 6

48

22

LEVO

PI

313 [M+H]

+

109

83 ± 19

64

27

185

45 ± 8

64

20

245

100

64

18

MPROG

PI

345 [M+H]

+

97

26 ± 4

74

23

123

100

74

25

NOR

PI

299 [M+H]

+

91

85 ± 16

69

35

109

100

69

26

PROG

PI

315 [M+H]

+

97

98 ± 17

65

20

109

100

65

24

^a^ The most abundant product ion was used for quantification whereas the other product ions were used for to confirm the presence of the steroid hormones.

**References**

1. Beuhler RJ, Flanigan E, Greene LJ, Friedman L (2002): **Proton transfer mass spectrometry of peptides. Rapid heating technique for underivatized peptides containing arginine**. J Am Chem Soc, **96:** 3990-3999.

2. Dams R, Benijts T, Duff K, Bell D (2002): **Influence of the eluent composition on the ionization efficiency for morphine of pneumatically assisted electrospray, atmospheric-pressure chemical ionization and sonic spray**. Rapid Commun Mass Spectrom, **16:** 1072-1077.

3. Daves GD (1979): **Mass spectrometry of involatile and thermally unstable molecules**. Acc Chem Res, **12:** 359-365.

4. DrugBank Open Data Drug and Drug Target Database. <http://www.drugbank.ca> . (accessed August 2012). 2012.

5. Geerdink RB, Kooistra-Sijpersma A, Tiesnitsch J, Kienhuis PGM, Brinkman UAT (1999): **Determination of polar pesticides with atmospheric pressure chemical ionisation mass spectrometry-mass spectrometry using methanol and/or acetonitrile for solid-phase desorption and gradient liquid chromatography**. J Chrom A, **863:** 147-155.

6. Harrison HG (1983): *Chemical Ionization Mass Spectrometry*. CRC Press Inc, Boca Raton, FL. pp. 156

7. Interactive PhysProp Database Demo. <http://www.syrres.com> . (accessed August 2012). 2012.

8. Ma YC and Kim HY (1997): **Determination of Steroids by Liquid Chromatography/Mass Spectrometry**. J Am Soc Mass Spec, **8:** 1010-1020.

9. Matuszewski BK, Constanzer ML, Chavez-Eng CM (2003): **Strategies for the Assessment of Matrix Effect in Quantitative Bioanalytical Methods Based on HPLC−MS/MS**. Anal Chem, **75:** 3019-3030.

10. Picard, Pierre, Tremblay, Patrice, and Paquin, E. R. Laser Diode Thermal Desorption Ionization Source (LDTD): Fundamental Aspects. 56th ASMS Conference on Mass Spectrometry. 2008.

11. Sinha V, Custer TG, Kluepfel T, Williams J (2009): **The effect of relative humidity on the detection of pyrrole by PTR-MS for OH reactivity measurements**. Int J Mass Spectrom, **282:** 108-111.

12. Tani A, Hayward S, Hansel A, Hewitt CN (2004): **Effect of water vapour pressure on monoterpene measurements using proton transfer reaction-mass spectrometry (PTR-MS)**. Int J Mass Spectrom, **239:** 161-169.

13. Zook DR and Grimsrud EP (1988): **Ion-Molecule Reactions in API**. J Phys Chem, **92:** 6374-6378.
